# Placental response to maternal SARS-CoV-2 infection

**DOI:** 10.1038/s41598-021-93931-0

**Published:** 2021-07-13

**Authors:** Mirella Mourad, Taylor Jacob, Elena Sadovsky, Shai Bejerano, Glicella Salazar-De Simone, Tarique Rajasaheb Bagalkot, Jason Zucker, Michael T. Yin, Jennifer Y. Chang, Lihong Liu, Larisa Debelenko, Carrie J. Shawber, Morgan Firestein, Yingshi Ouyang, Cynthia Gyamfi-Bannerman, Anna Penn, Alexander Sorkin, Ronald Wapner, Yoel Sadovsky

**Affiliations:** 1 Department of Obstetrics, Gynecology and Reproductive Science, College of Physicians and Surgeons, Columbia University Irving Medical Center, New York, NY USA; 2 Magee-Womens Research Institute, Department of Obstetrics, Gynecology and Reproductive Sciences, University of Pittsburgh, 204 Craft Avenue, Pittsburgh, PA 15213 USA; 3 Department of Cell Biology, University of Pittsburgh School of Medicine, Pittsburgh, PA USA; 4 Department of Medicine, College of Physicians and Surgeons, Columbia University Irving Medical Center, New York, NY USA; 5 Department of Pathology and Cell Biology, College of Physicians and Surgeons, Columbia University Irving Medical Center, New York, NY USA; 6 Department of Surgery, College of Physicians and Surgeons, Columbia University Irving Medical Center, New York, NY USA; 7 Department of Psychiatry, College of Physicians and Surgeons, Columbia University Irving Medical Center, New York, NY USA; 8 Department of Pediatrics, College of Physicians and Surgeons, Columbia University Irving Medical Center, New York, NY USA; 9 Department of Microbiology and Molecular Genetics, University of Pittsburgh School of Medicine, Pittsburgh, PA USA

**Keywords:** SARS-CoV-2, Viral infection

## Abstract

The coronavirus disease 2019 (COVID-19) pandemic affected people at all ages. Whereas pregnant women seemed to have a worse course of disease than age-matched non-pregnant women, the risk of feto-placental infection is low. Using a cohort of 66 COVID-19-positive women in late pregnancy, we correlated clinical parameters with disease severity, placental histopathology, and the expression of viral entry and Interferon-induced transmembrane (IFITM) antiviral transcripts. All newborns were negative for SARS-CoV-2. None of the demographic parameters or placental histopathological characteristics were associated with disease severity. The fetal-maternal transfer ratio for IgG against the N or S viral proteins was commonly less than one, as recently reported. We found that the expression level of placental *ACE2,* but not *TMPRSS2* or *Furin,* was higher in women with severe COVID-19. Placental expression of IFITM1 and IFITM3, which have been implicated in antiviral response, was higher in participants with severe disease. We also showed that IFITM3 protein expression, which localized to early and late endosomes, was enhanced in severe COVID-19. Our data suggest an association between disease severity and placental SARS-CoV-2 processing and antiviral pathways, implying a role for these proteins in placental response to SARS-CoV-2.

## Introduction

The severe acute respiratory syndrome coronavirus 2 (SARS-CoV-2), which is responsible for the coronavirus disease 2019 (COVID-19) pandemic, has been a major threat to populations worldwide. With nearly 150 million annual births worldwide, the risk to pregnant women is significant. Whereas the percentage of pregnant women infected by SARS-CoV-2 might be similar to that of non-pregnant women, the impact of COVID-19 on gravid symptomatic women has been greater than on age-matched non-pregnant women, with more frequent admissions to intensive care units, dependence on ventilatory and circulatory support, and even deaths^1–4^. This increased risk of maternal complications is reminiscent of the risk from influenza or other SARS epidemics^5–7^.

Although the upper and lower airways are “ground zero” for attack by the airborne SARS-CoV-2, viremia is found in 8–40% of the patients with COVID-19^8^ and leads to extrapulmonary manifestations affecting multiple organs^9^. Because nearly 25% of the cardiac output of pregnant mothers in the latter part of pregnancy perfuses the utero-placental unit, the risk of SARS-CoV-2 spread to the feto-placental unit is substantial. Yet, in the majority of pregnant women with confirmed COVID-19, the placenta and fetus were found to be uninfected^2,10–17^, with current estimates of feto-placental transmission of SARS-CoV-2 near 2–3%^10^. Notably, there have been isolated or clustered case reports that detailed placental infection, with and without fetal involvement, with a range of accuracy and reliability that reflect the methods used for virus detection, affected tissue-types, and the timing of infection relative to maternal infection^18–28^. Thus, although reports suggest that the spread to the feto-placental unit is more common in symptomatic, severe COVID-19 infections and in women with underlying medical or obstetrical diseases, these data might have been affected by ascertainment bias and a lack of broad, prospective testing of pregnant women across pregnancy.

The recognition of the single-stranded, enveloped SARS-CoV-2 by target cells and the subsequent viral cell entry requires an interaction of the viral spike (S) protein with ACE2 and other proteins (such as CD147, DPP4, GRP78, L-SIGN, and DC-SIGN) that may facilitate viral binding^29–34^. Subsequent processing is carried out by the serine protease TMPRSS2 and the endosomal protease furin^35–38^. Although initial reports regarding human placental expression of key SARS-CoV-2 entry proteins have been inconsistent^39,40^, the expression of these proteins in placental trophoblasts has been subsequently validated^29,36,41–47^. In this work, we used prospectively collected placental biopsies to assess the effect of COVID-19 severity on the expression of SARS-CoV-2 entry factors ACE2, TMPRSS2, and furin. We also assessed placental expression of Interferon-induced transmembrane (IFITM) proteins, which are a part of innate immune sensing. IFITM proteins are expressed in epithelial cells and protect them from coronavirus replication^48–51^.

## Results

### Participant characteristics and maternal-neonatal outcomes

**Figure 1:**
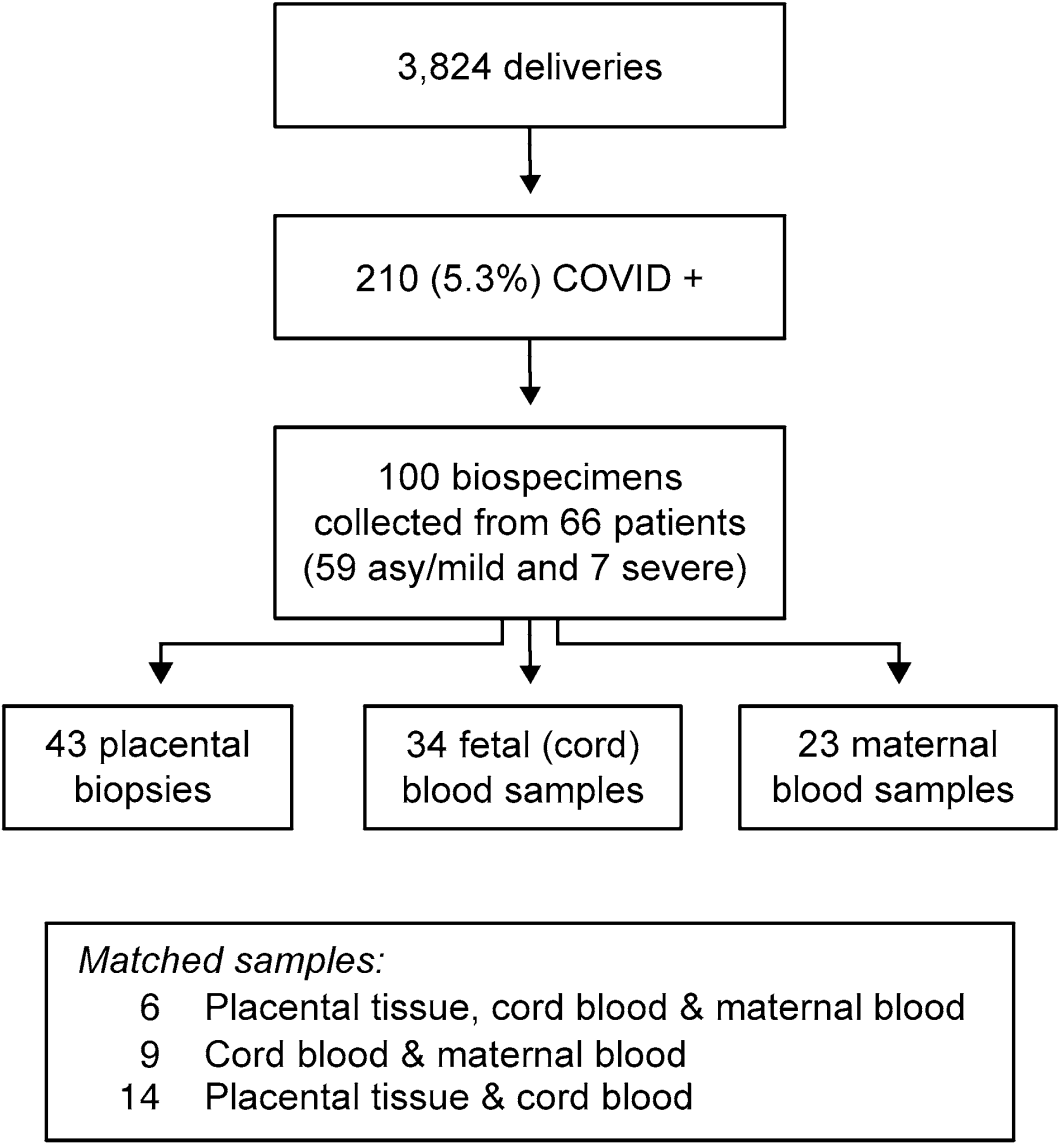
Study participants and specimens collected for the study. See text for details. Matched maternal-placental-fetal samples were collected from a small fraction of the patients.

We included in this study 66 COVID-19-positive women: 59 were either asymptomatic or had mild COVID-19, and 7 had severe disease (Fig. 1). As shown in Table 1, women in the asymptomatic/mild disease group were younger than women in the severe group (mean age at delivery for all participants 28.6 ± 5.8 *vs* 36.0 ± 4.7 years, respectively, p = 0.005). The majority of participants (73%) identified their ethnic origin as Hispanic/Latino/Spanish, with a distribution of other ethnic groups as shown in Table 1. Pre-pregnancy BMI, reported for 44/66 participants, and the incidence of key maternal medical diseases were not significantly different between the groups. The median gestational age at COVID-19 detection and the gestational age at delivery were later for the asymptomatic/mild group when compared to the severe disease group (Tables 1, 2), although the time from symptom onset or disease detection to delivery was insignificantly different (Table 2).

**Table 1:** Maternal demographics, based on the severity of COVID 19 symptoms.

	Asymptomatic/mild (n = 59)		Severe (n = 7)		p-value
Maternal age (years, n = 66)	28.64 ± 5.75		36.0 ± 4.69		0.0005
**Race (n = 66)**					
Hispanic	43	(72.9%)	5	(71.4%)	0.54
Asian	2	(3.4%)	0		
Black	2	(3.4%)	1	(14.3%)	
White	8	(13.6%)	1	(14.3%)	
Unknown/not specified	4	(6.8%)	0		
BMI (n = 44)	29.32 ± 6.06		30.80 ± 4.27		0.56
**Medical conditions (n = 66)**					
Asthma	9	(15.3%)	2	(28.6%)	0.33
Chronic hypertension	3	(5.1%)	1	(14.3%)	0.37
Gestational diabetes	8	(13.6%)	2	(28.6%)	0.29
Gestational age at diagnosis (n = 65)	38	[35.86 – 39.43]	35.86	[30.43 – 37.00]	0.014

*The categorical values are expressed as n (%), the continuous variables are expressed as either median [IQR] or mean ± SD.

**Table 2:** Maternal clinical outcomes, based on severity of COVID 19 symptoms.

	Asymptomatic/mild (n = 59)		Severe (n = 7)		p-value
Gestational age at delivery (n = 66)	39	[37.71 – 40.0]	36.3	[35.29 – 37.14]	0.006
**Mode of delivery (n = 66)**					
Cesarean section	25	(42.4%)	5	(71.4%)	0.23
Vaginal delivery	34	(57.6%)	2	(28.6%)	
Length of time from symptom onset to delivery (days, n = 36)	20	[12.0–41.0]	10	[6.0–35.0]	0.50
Length of time from detection to delivery (days, n = 65)	1	[0–12]	7	[0–31]	0.23
**COVID-19 related therapies (n = 66)**					
Hydroxychloroquine	3	(5.1%)	5	(71.4%)	< 0.001
Remdesivir	0		3	(42.9%)	< 0.001
Tocilizumab	0		1	(14.3%)	0.11
Other interleukin inhibitor	0		1	(14.3%)	0.11
Azithromycin	2	(3.4%)	2	(28.6%)	0.053
IV antibiotics	1	(1.7%)	7	(100%)	< 0.001
Oxygen requirement (n = 66)	1	(1.7%)	6	(85.7%)	< 0.001
Mechanical ventilation (n = 66)	0		2	(28.6%)	0.01

*The categorical values are expressed as n (%), the continuous variables are expressed as either median [IQR] or mean ± SD.

The maternal outcomes, analyzed by the severity of COVID-19, are presented in Table 2. There were no differences in the mode of delivery. As expected, the use of oxygen and several COVID-19 medications and even the use of IV antibiotics were more common in the severe COVID-19 group. The neonatal outcomes are shown in Table 3 and revealed no differences with respect to neonatal sex, Apgar scores, birth weight, or the rate of NICU admission. The reasons for NICU admission were related to complications of prematurity. All newborns in our study tested negative for SARS-CoV-2.

**Table 3:** Neonatal clinical outcomes, based on severity of maternal COVID-19 symptoms.

	Asymptomatic/mild (n = 59)		Severe (n = 7)		p-value
**Sex** (n = 66)					
Male	23	(39.0%)	3	(42.9%)	> 0.99
Female	36	(61.0%)	4	(57.1%)	
**Apgar scores (n = 65)**					
Apgar score 1	9.0	[8.0–9.0]	9	[8.0–9.0]	0.90
Apgar score 5	9.0	[9.0–9.0]	9	[9.0–9.0]	> 0.99
Birthweight (n = 66)	3125.36 ± 622.56		2692.29 ± 677.86		0.15
Admission to the NICU (n = 65)	9	(15.5%)	2	(28.6%)	0.34
**COVID-19 test (+ /–, n = 66)**					
Not detected	58	(98.3%)	7	(100%)	> 0.99
Indeterminate or detected	1	(1.7%)	0		
Not done	0		0		
Neonatal demise (n = 66)	0		0		
Fetal demise (n = 66)	1	(1.7%)	0		> 0.99

*The categorical values are expressed as n (%), the continuous variables are expressed as either median [IQR] or mean ± SD.

### Placental histopathology

The histopathological features of the placenta, analyzed on the basis of maternal COVID-19 severity, are presented in Table 4. Of the 66 participants, 59 had placental histopathology analysis performed for clinical indications, including all 7 severely symptomatic patients. There were no differences between the two groups with respect to the frequency of maternal vascular malperfusion (MVM) lesions, fetal vascular malperfusion (FVM) lesions, acute inflammatory processes, chronic inflammatory processes, or lesions categorized as not fitting into these groups. Placental histopathologic result of the two cases that were placenta-positive for SARS-CoV-2 by PCR (one with severe diseases and one asymptomatic) is provided in Supplementary Table 2.

**Table 4:** Analyses of histopathology of placentas (n = 59).

	Mild/asymptomatic (n = 52) n/(%)		Severe (n = 7) n/(%)		p-value
MVM	2	(3.8%)	0		> 0.99
MVM-i*	26	(50.0%)	3	(42.9%)	> 099
FVM	8	(15.4%)	0		0.58
FVM-i*	29	(55.8%)	2	(28.6%)	0.24
AIP	12	(23.1%)	1	(14.3%)	> 0.99
CIP	6	(11.5%)	0		> 0.99
Other	20	(38.5%)	2	(28.6%)	0.7

i*Implies that the sample had one or more individual diagnostic features, but criteria for full diagnosis were not met.

### Plasma immune and cytokine profile

**Figure 2:**
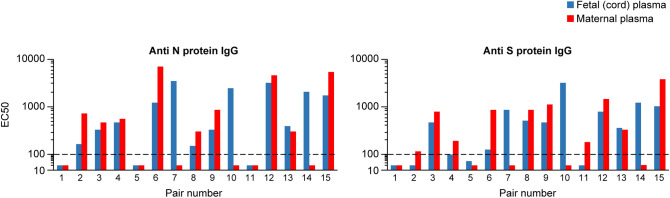
The level of IgG to the SARS-CoV2 N- or S-timer protein. Analysis was performed in 15 maternal–fetal paired plasma samples (x-axis), all with asymptomatic/mild disease. The y-axis denotes EC50 for each antibody, presented using a log10 scale. The level of detection was determined to be a 100-fold plasma dilution based upon previous studies. See text for details.

We were able to obtain maternal and fetal (cord) plasma pairs on 15 participants, all with asymptomatic/mild disease (Fig. 2). We examined the titers of anti SARS-CoV-2 S- and N-protein IgG, IgM, or IgA antibodies for the presence of systematic maternal–fetal differences. As expected, the fetal plasma samples were negative for IgM or IgA. Although the numbers were small, we noted that, for N-protein, the IgG transfer ratio, defined as the fetal (cord plasma) IgG concentration divided by maternal IgG plasma concentration^52^, was > 1 in 4 dyads and < 1 in 9 dyads. For S trimer, the values were > 1 in 4 dyads and < 1 in 10 dyads. We also determined the level of selected cytokines in the plasma of 34 women. Two of the 34 had severe COVID-19. As shown in Supplementary Table 1, there were no clear patterns of maternal cytokine levels that were characteristic of the two cases with severe COVID-19. Interestingly, we also found no association between cytokine levels and either the clinical characteristics (Tables 1, 2) or the placental histopathology (Table 4) samples for asymptomatic/mild COVID-19 cases (data not shown).

### Placental expression of SARS-CoV-2 entry factors and IFITM

**Figure 3:**
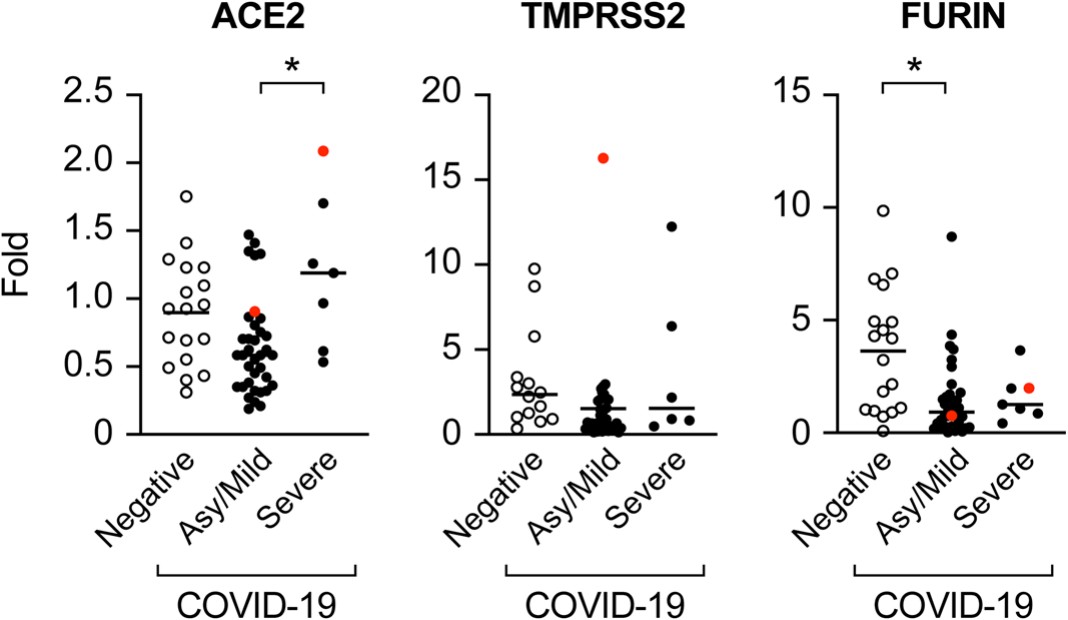
Placental expression of mRNAs for SARS-CoV-2 entry proteins. Analysis, stratified by disease severity (asymptomatic/mild *vs* severe) and negative controls, was performed using RT-qPCR as detailed in “Methods”. The red dot denotes SARS-CoV-2-positive placenta. The data were analyzed using the Kruskal–Wallis nonparametric test, with post hoc Tukey test for all pairwise comparisons. *Denotes p < 0.01.

Placental biopsies were obtained from 61 participants. We were able to extract suitable total RNA from 83% of the samples processed in RNAlater and 55% of the samples processed in FFPE (see “Methods”), resulting in suitable RNA samples from 43 participants with asymptomatic/mild (n = 36) or severe (n = 7) COVID-19. Eighteen additional biopsies were obtained from placentas of COVID-19-negative women, as described in “Methods”. In the absence of adequate specimens for protein analysis, all gene expression measurements were performed using RNA samples, analyzed separately for RNAlater- and FFPE-preserved samples. Using primers specific for the SARS-CoV-2 S- and N-sequences, which we showed to detect ≥ 8 viral RNA molecules (see “Methods”), we detected the presence of SARS-COV-2 in the placenta of two participants, one with asymptomatic/mild and one with severe COVID-19. Using all suitable RNA samples, we measured placental expression of mRNAs for SARS-CoV-2 entry factors, namely *ACE2*, *TMPRSS2*, and *furin*. As shown in Fig. 3, we found that *ACE2* levels were lower in asymptomatic/mild participants compared to those with severe disease. In contrast, we detected no difference in the expression level of *TMPRSS2* among participants in the three groups. The expression of furin was lower in participants with asymptomatic/mild disease compared to COVID-19-negative controls.

**Figure 4:**
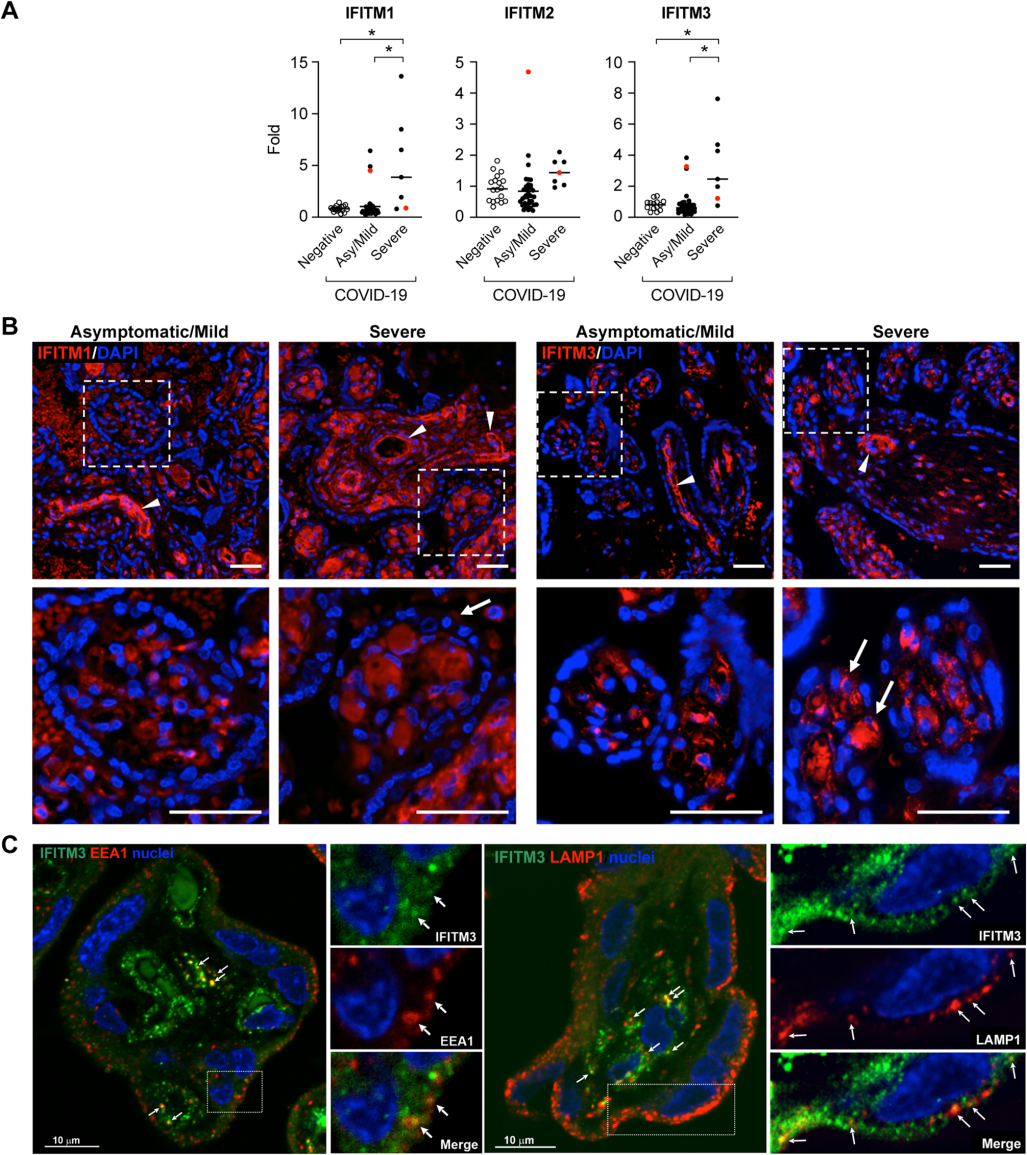
Placental expression of antiviral IFITM proteins. (**A**) Placental expression of IFITM1–3 mRNAs. Analysis, stratified by disease severity (asymptomatic/mild *vs* severe) and negative controls, was performed using RT-qPCR as detailed in “Methods”. The red dot denotes SARS-CoV-2-positive placenta. The data were analyzed using Kruskal–Wallis nonparametric test, with post hoc Tukey test for all pairwise comparisons. *Denotes p < 0.01. (**B**) Placental sections from participants with asymptomatic/mild *vs* severe COVID disease, stained for either IFITIM1 or IFITM3 immunofluorescence as described in “Methods”, and nuclei visualized with DAPI. Boxed areas are enlarged below. White arrowheads mark positive fetal stem villous vessels. White arrows mark rare less frequent trophoblast staining in terminal villi. Scale Bars: 50 µm. Negative-control immunofluorescence stains are shown in Supplementary Fig. 1. (**C**) Placental sections from negative-control term placentas, stained for IFITM3 immunofluorescence (green) and co-stained for either EEA1, a marker of early endosome, or LAMP1, a marker of late endosome/lysosome, as described in “Methods”. Nuclei were visualized with DAPI. Boxed areas are enlarged to the right side of each image. White arrowheads denote colocalization of IFITM3 and endosomal markers. Scale Bar: 10 µm.

IFITM are innate immune response genes^53^ that are expressed in the placenta and are known to limit viral infections^49,54,55^. Located at the cell membrane and endosomes, IFITM proteins can diminish viral fusion with the cell membrane or the fusion of viruses with late endosomal membranes for cytosolic entry^55^. Among the five human IFITM members, only IFITM1, IFITM2, and IFITM3 are known to be interferon responsive^55^. One of the members of this family, IFITM3, is an endosomal protein that was recently shown to restrict SARS-CoV-2 replication^56,57^. We found that placental mRNA expression of IFITM1 and IFITM3 was upregulated in participants with severe disease when compared to asymptomatic/mild COVID-19-positive pregnant women (Fig. 4A). Using immunofluorescence, we found that IFITM1 was localized within the villous core and around fetal capillaries, with weak expression in the trophoblast layer, and no difference was observed between women with asymptomatic/mild or severe disease. The expression of IFITM3 was similar to that of IFITM1, with signal enhancement in specimens from placentas of women with severe as opposed to asymptomatic/mild disease (Fig. 4B). We also showed that IFITM3 colocalized with EEA1 and LAMP1, markers of early and late endosomes, respectively (Fig. 4C). Finally, we found no correlation between the expression of SARS-CoV-2 entry factors and IFITM transcripts and placental histopathology (not shown).

## Discussion

Our clinical data corroborated previous observations, indicating that, on the basis of common definitions of infection^58^, fetal transmission is rare, even in women with severe COVID-19. A review of published histopathological analysis of placentas from pregnancies affected by COVID-19 revealed that an association with chronic histiocytic intervillositis was infrequently observed^18,19,24,27,28,59^. Whether or not there is a higher prevalence of maternal or fetal vascular malperfusion lesions remains controversial^28,46,60–62^. Whereas our data support the prevailing conclusion that there are no specific pathological lesions that characterize placentas from COVID-19-infected women, it is clear that reporting bias, lack of controls, and insufficient longitudinal analyses limits our ability to decipher the effect of SARS-CoV-2 infection on placental histomorphology.

Pertaining to the placental expression of SARS-CoV-2 entry proteins, we found increased ACE2 expression in women with severe *vs* asymptomatic/mild COVID-19. This change was not associated with increased fetal infection. The expression of TMPRSS2 was highly variable, but overall, similar among the groups. The expression of furin was reduced in placentas from women with asymptomatic/mild disease, when compared to negative controls. Our data are consistent with recent observations suggesting that the severity of clinical COVID-19 does not correlate with feto-placental transmission^63^.

The exact post-entry mechanism by which SARS-COV-2 interacts with host cell endocytic pathways remains to be explored. After endosomal processing, the viral genome is released to the cytosol, where it is translated into viral E, M, N, and S proteins, largely in the endoplasmic reticulum, promoting viral RNA replication^64^, with subsequent modifications in the Golgi. Although the remaining steps in SARS-COV-2 processing are not entirely understood, recent data indicate that SARS-COV-2 can usurp lysosomal exit pathways after deacidifying the lysosome’s content and deactivating its enzymes^65^. Several interferon-stimulated genes were shown to affect SARS-CoV-2 replication. These include the lymphocyte antigen 6 complex locus E (LY6E), which interferes with SARS-CoV-2 entry into cells by attenuating S-protein-mediated membrane fusion^56,66^ and the zinc-finger antiviral protein (ZAP), which limits SARS-CoV-2 replication^67^. In this work, we focused on the evolutionarily conserved IFITM proteins, which are expressed in epithelial cells, including placental trophoblasts, where they restrict the replication of influenza A, SARS-CoV-1, flaviviruses, and several other enveloped viruses^49,50,54,68–71^. Unlike other interferon-inducible proteins that interfere with viral replication, IFITM proteins were shown to block membrane fusion of enveloped viruses by using an amphipathic helix domain to alter membrane lipid order and curvature, and inhibit virus-induced fusion pore formation^49^. Notably, in certain cases, IFITM proteins have also been shown to promote cell membrane entry by coronavirus species^48,57,72,73^. Interestingly, the antiviral function of IFITM3 may be attenuated by overexpressed TMPRSS2^56,57^, yet we observed no change in TMPRSS2 expression.

In the human placenta, a homozygous SNP rs12252-C mutation in IFITM3 predisposes to SARS-CoV-1 and SARS-CoV-2 infection^71,74,75^. Among IFITM proteins, IFITM3 is also known to have the greatest effect on reducing trophoblast fusion^76,77^. We showed that the expression of placental *IFITM1* and *IFITM3* transcripts was upregulated in women with severe infection when compared to women with asymptomatic/mild disease or to negative controls. While the expression of IFITM1 and IFITM3 was prominent in the villous core and perivascular regions, IFITM3 was also localized to trophoblasts, and was upregulated in women with severe COVID-19. We also showed that IFITM3 colocalized to early and late endosomes, as shown in other cell systems^70^. Taken together, our data suggest that, in women with severe COVID-19, IFITM1 and IFITM3 proteins participate in trophoblastic immune response and possibly promote placental protection against SARS-CoV-2.

The transport of IgG from the maternal to the fetal circulation is facilitated by the trophoblastic FcRn receptor, usually leading to a higher concentration of IgG in the fetal than in the maternal circulation^78–84^. Our observation that, in the majority of maternal–fetal dyads, the transfer ratio for anti-NP and for anti-S-trimer IgG  is < 1 is consistent with recent data from Atyeo et al.^85^ and Edlow et al.^17^, who showed that altered glcosylation status of Fc and bias against the glycoforms of SARS-CoV-2 antibodies reduced IgG transport in maternal–fetal dyads with third trimester COVID-19.

Our work has several limitations. First, our sample size is relatively small, likely reflecting the milder nature of the disease in pregnancy. Second, we recognize that the definitions used for mild or severe COVID-19 are somewhat arbitrary and vary among investigators. We used a common definition that has also been used in reference to pregnant women^12,86–88^. Third, we had no access to well-preserved placental specimens in order to validate our data using western immunoblotting. We have highlighted the inconsistent manner (RNAlater and FFPE) used for preservation of our placental specimens. Indeed, we rejected samples on the basis of RNA quality, likely reflecting extended time between delivery and tissue acquisition. Fourth, all participants in our study were enrolled late in pregnancy, yet the initial infection might have occurred weeks earlier, resulting in a sampling bias. Considering the rapid changes in trophoblast development during pregnancy, conclusions cannot be extrapolated to women infected early in pregnancy. Notably, universal serology testing was not available before July 2020, limiting our ability to confirm the participants’ infection status earlier in the pregnancy. Lastly, although our analysis was not contingent upon placental infection status, we recognize that additional measures are needed to validate SARS-CoV-2 infection in the placenta. Nonetheless, our findings add to our knowledge regarding the repertoire of placental cell defense mechanisms^89–91^ and, specifically, to placental resistance to SARS-CoV-2 infection^27^.

## Methods

### Participants and specimen procurement

This prospective study included women who presented for labor and delivery at Columbia University Irving Medical Center (CUIMC) between March and October 2020 who tested positive for SARS-CoV-2 by way of PCR assay of nasopharyngeal swab^92^. Women diagnosed with SARS-CoV-2 were classified as asymptomatic/mildly symptomatic women or severely symptomatic on the basis of their symptoms and clinical findings as defined by the National Institutes of Health for non-pregnant adults and adopted by the Society of Maternal Fetal Medicine after modifications that take gestational physiology into account^93^. All participants with COVID-19 received care by the high-risk obstetrical team and neonatal teams at CUIMC. All newborns of SARS-CoV-2-positive mothers were tested for the virus by PCR, using nasopharyngeal swabs in the first 24 h of life. Negative PCR control samples were obtained from both CUIMC and the Steve N. Caritis Magee Obstetric Maternal & Infant (MOMI) Database and Biobank at Magee-Womens Research institute.

The study was approved by the institutional review boards at Columbia University Irving Medical Center (CUIMC, IRB # AAAT0191) and at the University of Pittsburgh (MOMI Databank, STUDY19100240), and all experiments were performed in accordance with relevant guidelines and regulations. All CUIMC’s COVID-19 biospecimens were procured and stored at the Columbia University Biobank (CUB), a centralized resource that coordinates the processing, storing, and dissemination of specimens for use in clinical research. Given the time sensitivity of obtaining the samples and the minimal risk to the patient, a deferred consenting model was implemented^94^. All postpartum patients who delivered within the New York Presbyterian Hospital system were contacted after discharge and were offered the chance to participate in the CUB. They provided written informed consent to have their tissues and/or blood stored with the CUB and made available for research. For the purposes of this study, specimens from eligible women were then requested from the CUB. Demographic information was collected, including maternal COVID-19 symptoms and related treatment and obstetric complications. All pregnant women who delivered at UPMC Magee-Womens Hospital in Pittsburgh provided written informed consent to participate in the MOMI Databank for the collection of data and biological specimens for use in biomedical research, under a protocol approved by the University of Pittsburgh.

Blood and placental biopsy specimens were obtained at delivery, whenever feasible, on the basis of clinical conditions and team availability. Maternal and fetal (cord) blood samples were collected from residual clinical samples that were obtained by the Columbia University Biobank. A 0.5^3^ cm placental biopsy, performed at a lesion-free midportion of the placenta that was equidistant from cord insertion and periphery^95^, was obtained by the clinical team at the time of delivery. Each specimen was placed in a 15-ml tube containing RNAlater (Invitrogen, Carlsbad, CA) and stored at 4 °C for 24–48 h before freezing at − 80 °C. Some samples were processed for formalin fixation and paraffin embedding (FFPE). Placental histopathological analysis was performed, as routinely, by CUIMC’s Department of Pathology and Cell Biology, and results were obtained from the health records of consented participants. Placental biopsies from healthy, COVID-19-negative term pregnant women included samples in either RNAlater or FFPE sections.

### Maternal serum and fetal cord blood antibody and cytokine analysis

The level of selected cytokine (IL10, IL17A, IL1β, IL6, IP10, MCP1, MIP1β, TNFα, IL28A, IL28β, and IL29) in the plasma or serum samples were measured using Milliplex human cytokine/chemokine magnetic bead panels (Millipore Sigma, St. Louis, MO) and the Luminex 200 platform (Luminex, Austin, TX). The samples were processed according to the manufacturer’s instructions, and cytokine concentrations were quantitated by Luminex xPONENT v3.1 and MILLIPLEX Analyst v5. The intra- and inter-assay precision of these cytokines varied between 1.6–4.4% and 6.7–18.3%, respectively. The mean intra-assay accuracy was 97%.

Immunoassays were used to quantify plasma antibodies to SARS-CoV-2 S trimer and nucleocapsid protein (NP) as previously described^96,97^. Briefly, SARS-CoV-2 spike trimer, or NP were coated on 96-well ELISA plate at a concentration of 50 ng/well, respectively at 4 °C overnight. After washing with 0.05% Tween-20 in PBS (PBST), plates were blocked with 300 μl/well of blocking buffer (1% BSA and 10% bovine calf serum in PBS) for 1 h at 37 °C, then washed again with PBST. Antibodies or heat-inactivated plasma samples from COVID-19 patients or healthy donors were serially diluted in a buffer (1% BSA and 20% bovine calf serum in PBS) and then incubated in the plates for 1 h at 37 °C. The plates were then washed with PBST and incubated with Peroxidase AffiniPure goat anti-human IgG (H + L) and goat anti-human IgM antibodies (cat #109-035-003 and 109-035-043, Jackson ImmunoResearch, West Grove, PA, both at 1:10,000 dilution) for 1 h at 37 °C. After a final PBST wash, the antibody binding was detected by incubating with Tetramethylbenzidinsubstrate (Sigma-Aldrich, St. Louis, MO, cat #4444) for 3 min. The reaction was stopped with 1 N sulfuric acid (cat# SA212-1, Thermo Fisher, Waltham, MA). Absorbance was measured at 450 nm and the OD450 values were analyzed using GraphPad Prism 8 (GraphPad, San Diego, CA).

### RNA extraction and RT-quantitative PCR

RNA was extracted from placental biopsies in RNAlater using TRI reagent (Molecular Research Center, Cincinnati, OH). The RNA was purified using EconoSpin spin columns (Epoch Life Science, Missouri City, TX). Extracted RNA samples were exposed to RNase-free DNase (Qiagen, Germantown, MD) according to the manufacturer's instructions. RNA was extracted from FFPE samples, using Qiagen’s RNeasy FFPE kit (cat #73504) and deparaffinization solution (cat #19093). The quantity and quality of total RNA was determined by a NanoDrop 1000 spectrometer (Thermo Fisher), and selected samples were validated using the Agilent bioanalyzer (Agilent, Santa Clara, CA).

Reverse transcription and quantitative PCR (RT-qPCR) was performed in duplicate, using the ViiA 7 Sequence Detection System (Thermo Fisher) as previously described^98^. For mRNA analysis, total RNA was reverse transcribed using the High-Capacity cDNA Reverse Transcription kit (Thermo Fisher) according to the manufacturer’s protocol. Quantitative PCR was performed by means of SYBR Select (Thermo Fisher). For miRNA, cDNA synthesis and qPCR were performed with the miRScript PCR system (Qiagen) according to the manufacturer's protocols. PCR primers are given in Supplementary Table 3. Dissociation curves were run on all reactions. mRNA samples were normalized to the expression of the GAPDH. The fold increase relative to control samples was determined by the 2-ΔΔCt method^99^ and compared to SARS-CoV-2-negative controls and was performed separately for specimens that were preserved in RNAlater and those preserved in FFPE.

The presence of SARS-CoV-2 in the placental biopsies was determined using RT-qPCR. As a positive control, we used two lung tissue samples, obtained from two COVID-19 autopsies that were verified using PCR and in situ hybridization at CUIMC and processed in RNAlater. To assess the sensitivity of our PCR assay, we used synthetic RNA transcripts of SARS-CoV-2 N1 and N2 RT-PCR amplicon sequences (Bio-Synthesis, Lewisville, TX), diluted to 100,000 transcript copies/μl in RNA storage solution. This was further diluted to 1000 transcript copies/μl with extracted nucleic acid from human embryonic lung cells, and an extract was used as an internal negative control. We screened two primer sets (Supplementary Table 1) and found that the sensitivity of the N1 set was > 100-fold higher. Using serial dilutions, we determined that our N1 primer set detected the presence of ≥ 8 copies of SARS-CoV2 N1 at PCR cycle < 35. Notably, all positive samples were verified using the SARS-CoV-2 S-protein primers (Supplementary Table 1).

### Immunofluorescence staining

For IFITM1 and IFITM3 immunofluorescence, we used paraffin-embedded villous sections from six different placentas (asymptomatic/mild, n = 2, or severe, n = 2, and two negative controls) and stained them with an antibody for IFITM1 (Sigma, cat #HPA004810, 1ug/ml) or IFITM3 (Cell Signaling, Danvers, MA, cat #59212T lot #1, 4.9 ug/ml), detected with the proper secondary antibodies (Donkey Anti-Rabbit Alexa Fluor-594 antibody, Invitrogen, cat #A-21207), and mounted using Vectashield mounting media containing DAPI (Vector Laboratories, Burlingame, CA, cat #H-1200-10). Tissue processed without primary antibodies served as a negative control. Images were captured with an Olympus IX83 inverted microscope and Olympus cellSens software and adjusted using Adobe Photoshop. Expression relative to control was determined by two separate individuals reviewing unmodified images (n > 5 per sample) and scored for presence of strong signal versus weak or absent signal.

For co-staining of IFITM3 and endosomal proteins, cryosections of human placenta were labeled with polyclonal IFITM3 (as above, 2 μg/ml), rat monoclonal anti-lysosome-associated membrane protein 1 (LAMP1, University of Iowa Developmental Studies Hybridoma Bank (#1D4B, 2 μg/ml)^100^ and mouse monoclonal EEA1 antibodies (#610457, BD Bioscience, USA) (2 μg/ml). Secondary antibodies were anti-rabbit, anti-rat, and anti-mouse AffiniPure antibodies conjugated with AlexaFluor-488 (A488), Cy5, and Cy3, respectively (Jackson Immuno Research). Nuclei were stained with Hoechst 33342 (Thermo Fisher, #62249). Co-localization of IFITM3 with endolysosomal markers was determined using a spinning disk confocal microscope system as described previously^47^ by identifying clear overlapping structures that could be followed in multiple z-planes.

### Statistics

The analysis first focused on the association of COVID-19 symptom severity (asymptomatic/mild *vs* severe) with maternal baseline demographic variables, maternal and neonatal clinical outcomes, and histopathology variables. Next, cytokine/antibody associations (classified as high expression *vs* low/negative cytokine expression, and antibodies as modest *vs* weak/negative expression) with clinically significant maternal baseline demographic variables and histopathology variables were tested. Observations with missing data were excluded from the relevant analyses. Fisher’s exact test was used to analyze categorical and binary variables, and Welch’s t-test or exact Wilcoxon-Mann–Whitney test used to analyze continuous variables, as appropriate. Ordinal variables, such as Apgar scores, were also analyzed by exact Wilcoxon–Mann–Whitney test. Normality was assessed via Q-Q plots and histograms. For variables that had a very small sample size for one of the exposure groups (< 10), it was assumed that the distributions of the two groups were similar, and normality was assessed primarily by the larger of the groups.

For the cytokine and antibody analyses, we had a relatively small number of participants, mostly from the asymptomatic/mild COVID-19 group. We thus elected a priori to use Fisher’s exact test and the exact Wilcoxon–Mann–Whitney test for categorical and continuous variables, respectively, and to only include the participants from the asymptomatic/mild COVID-19 group. Categorical variables for antibody levels (modest, weak, negative) were based upon classifications determined in previous studies using the same assay we previously detailed^96,97^. Categorical variables for cytokine levels were based upon levels above and below the median (high and low, respectively) for each cytokine, based upon available data from the manufacturer. A modified Bonferroni correction was used to adjust for multiple comparisons in the cytokine/antibody analyses. As the levels of different cytokine/antibody variables may be correlated, their tests may also be correlated. To calculate the effective number (M_eff_) of independent tests, we used the method proposed by Li et al.^101^ to obtain a new significance level of 0.05/M_eff_. This adjustment to the alpha level was applied to maternal and cord blood cytokine and antibody variables separately. Otherwise, the alpha level was equal to 0.05. All analyses were two-sided and conducted using RStudio version 1.2.5042 (RStudio, Inc., Boston, MA, USA) or SAS version 9.4 (SAS Institute, Inc., Cary, NC, USA). For RNA analysis, fold-change data were analyzed using the Kruskal–Wallis nonparametric test, with post hoc Tukey test for all pairwise comparisons. Analyses were performed using Prism software (GraphPad).

## Supplementary Information

Supplementary Information.
