# Supplementary material for: Placental response to maternal SARS-CoV-2 infection
Source: Sci Rep. 2021 Jul 13;11:14390. doi: 10.1038/s41598-021-93931-0 (PMC8277865; doi:10.1038/s41598-021-93931-0)
Supplement: Supplementary file 1 — Supplementary Information. [file 41598_2021_93931_MOESM1_ESM.pdf]

SUPPLEMENTARY MATERIAL: Placental response to maternal SARS-CoV-2 infection

Authors: Mirella Mourad, MD, Taylor Jacob, PhD, Elena Sadovsky, Shai Bejerano, MS, Glicella Salazar-De Simone, PhD, Tarique Rajasaheb Bagalkot, PhD, Jason Zucker, MD, Michael T. Yin, MD, Larissa Debelenko, MD, PhD, Carrie J. Shawber, PhD, Morgan Firestein, PhD, Yingshi Ouyang, PhD, Cynthia Gyamfi-Bannerman, MD, Anna Penn, MD, PhD, Alexander Sorkin, PhD, Ronald Wapner, MD, Yoel Sadovsky, MD

This file contains 1 Supplementary Figure and 3 Supplementary Tables.

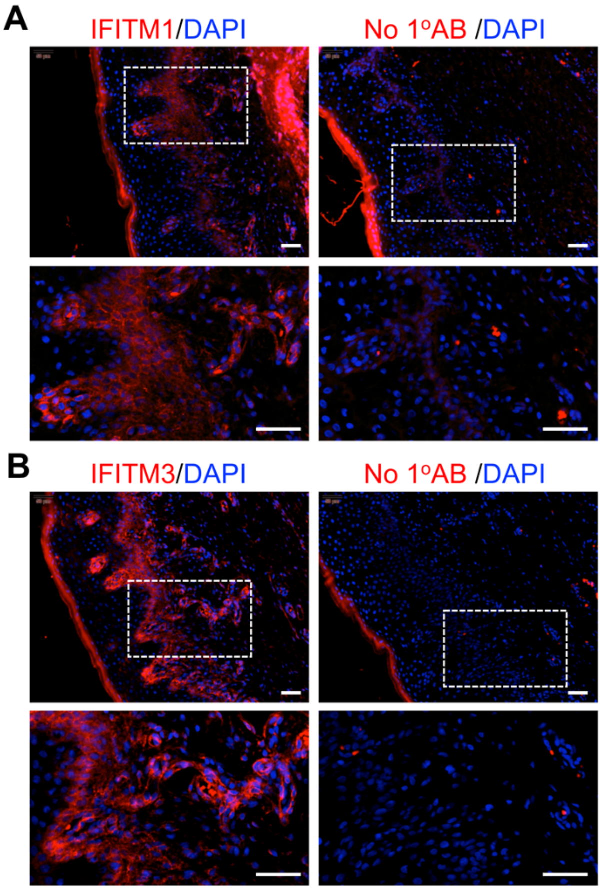

Supplementary Figure 1. IFITM1 and IFITM3 antibody validation. Paraffin-embedded human neonatal dermal tissue stained for either A) IFITM1 or B) IFITM3 (left panels) and compared to serial section without primary antibody (right panels). Boxed areas are enlarged below. Scale bar: 50 μm.

Supplementary Table 1. Cytokine data.

#	IL10 pg/ml	IL17A pg/ml	IL1b pg/ml	IL6 pg/ml	IP10 pg/ml	MCP1 pg/ml	MIP1b pg/ml	TNFa pg/ml	IL28A pg/ml	IL28B pg/ml	IL29 pg/ml
**1**	<3.20↓	<2.69↓	**10.95**	<2.93↓	287.38	134.31	59.94	**38.82**	<8.07↓	172.81	<368.70↓
**2**	3.47	<2.69↓	**12.79**	4.24	171.30	129.14	86.66	**31.55**	<8.07↓	172.81	<368.70↓
**3**	<3.20↓	<2.69↓	**9.99**	<2.93↓	175.51	**221.03**	63.97	18.25	<8.07↓	339.57	<368.70↓
**4**	<3.20↓	<2.69↓	**9.99**	3.71	84.23	<107↓	**90.35**	13.82	<8.07↓	166.90	<368.70↓
**5**	<3.20↓	<2.69↓	**14.85**	<2.93↓	**365.67**	163.51	**100.26**	**31.48**	<8.07↓		
**6**	<3.20↓	<2.69↓	4.73	<2.93↓	**350.71**	112.34	74.35	**30.33**	<8.07↓	**3964.00**	<368.70↓
**7**	<3.20↓	<2.69↓	**7.81**	<2.93↓	305.19	108.42	**119.76**	**34.44**	<8.07↓		<368.70↓
**8**	4.01	<2.69↓	<2.61↓	<2.93↓	210.63	**216.11**	<57.9↓	**21.46**	<8.07↓	390.40	<368.70↓
**9**	<3.20↓	<2.69↓	**8.56**	<2.93↓	233.23	206.02	68.96	**30.13**	<8.07↓		
**10**	4.29	<2.69↓	**7.76**	4.24	189.53	133.84	**142.95**	**23.47**	<8.07↓		
**11**	<3.20↓	<2.69↓	3.80	3.94	235.42	185.11	**121.97**	**40.70**	52.89		
**12**	4.76	<2.69↓	5.17	6.53	129.05	149.84	**95.03**	**25.42**	**179.12**	115.27	581.16
**13**	7.35	<2.69↓	3.51	<2.93↓	277.60	194.66	74.47	**32.36**	<8.07↓	149.35	<368.70↓
**14**	<3.20↓	<2.69↓	5.35	<2.93↓	126.97	177.39	77.32	17.72	<8.07↓	245.59	451.65
**15**	9.45	<2.69↓	2.67	<2.93↓	228.22	143.38	72.03	**36.87**	<8.07↓	104.27	<368.70↓
**16**	7.77	<2.69↓	<2.61↓	<2.93↓	230.38	174.04	75.58	**35.86**	<8.07↓		
**17**	5.05	<2.69↓	<2.61↓	<2.93↓	**1020.00**	136.99	**149.99**	**42.58**	<8.07↓	166.90	**791.04**
**18**	<3.20↓	<2.69↓	4.56	<2.93↓	**329.16**	203.71	**130.52**	**31.75**	<8.07↓		
**19**	6.54	<2.69↓	<2.61↓	<2.93↓	172.54	169.88	**116.41**	**26.83**	<8.07↓	172.81	623.74
**20**	4.20	<2.69↓	<2.61↓	<2.93↓	**531.71**	**494.45**	<57.9↓	17.72	<8.07↓	409.53	<368.70↓
**21**	5.54	<2.69↓	<2.61↓	23.02	**324.39**	**310.98**	<57.9↓	<5.7↓	**612.68**	415.91	**791.04**
**22**	227.70	67.15	4.60	**4983.00**	**1162.00**	**1233.00**	**91.21**	**38.42**	8.38	754.31	**749.66**
**23**	7.77	<2.69↓	<2.61↓	5.81	155.19	186.28	<57.9↓	9.43	**203.39**	239.42	495.11
**24**	**108.20**	<2.69↓	<2.61↓	**71.25**	**2481.00**	**960.96**	**210.39**	**79.57**	<8.07↓		
**25**	8.82	<2.69↓	<2.61↓	26.00	**325.70**	**289.21**	<57.9↓	11.22	<8.07↓	601.46	<368.70↓
**26**	14.05	<2.69↓	<2.61↓	3.37	**2600.00**	**380.88**	<57.9↓	16.79	<8.07↓	**1403.00**	<368.70↓
**27**	9.35	<2.69↓	<2.61↓	<2.93↓	229.00	<107↓	<57.9↓	9.88	65.64	722.58	**2220.00**
**28**	5.74	<2.69↓	<2.61↓	<2.93↓	244.59	**222.06**	70.48	10.19	<8.07↓	2992.00	<368.70↓
**29**	9.24	9.97	<2.61↓	28.60	**3410.00**	**1082.00**	<57.9↓	**29.59**	**502.90**	409.53	<368.70↓
**30**	**30.34**	4.46	**9.75**	**49.70**	**3764.00**	**485.24**	<57.9↓	**24.41**	**1489.00**	658.93	623.74
**31**	<3.20↓	<2.69↓	<2.61↓	<2.93↓	**452.42**	203.37	<57.9↓	10.64	<8.07↓	**1730.00**	<368.70↓
**32**	3.65	<2.69↓	<2.61↓	<2.93↓	**510.50**	**360.15**	<57.9↓	16.13	38.59	276.65	**913.44**
**33**	3.29	<2.69↓	<2.61↓	11.25	**388.93**	113.08	63.41	19.25	**281.86**	208.83	666.01
**34**	5.93	<2.69↓	<2.61↓	<2.93↓	**399.93**	**395.65**	<57.9↓	7.13	<8.07↓	333.24	<368.70↓

Supplementary Table 2. Histopathology of the two SARS-CoV-2 PCR-positive cases.

Clinical severity	Histopathology	SARS-CoV-2 staining	
		***in situ* hybridization**	**Immuno-histochemistry**
Severe	Mild acute inflammatory infiltrate in amnionic plate, increased decidual fibrin	Negative	Negative
Asymptomatic	Perivillous fibrin deposition, associated with chronic villitis and intervillositis, involving approximately 5-10%; partial myofibroblastic occlusion in chorionic vessels, consistent with fetal vascular malperfusion	Positive	Positive

Supplementary Table 3. Primer data.

PrimerQ#	Gene name	Gene full name	Direction	Sequence
Q6879Q6880	ACE2	Angiotensin I converting enzyme 2	F	CGAAGCCGAAGACCTGTTCTA
Q6877Q6878	TMPRSS2	Transmembrane protease serine 2	F	GTCCCCACTGTCTACGAGGT
Q6871Q6872	FURIN	FURIN	F	CCTGGTTGCTATGGGTGGTAG
			R	AAGTGGTAATAGTCCCCGAAGA
Q6749Q6750	IFITM1	Interferon induced transmembrane protein 1	F	CCAAGGTCCACCGTGATTAAC
			R	ACCAGTTCAAGAAGAGGGTGTT
Q6751Q6752	IFITM2	Interferon induced transmembrane protein 2	F	ATGAACCACATTGTGCAAACCT
			R	CGGAGTAGGCGAATGCTATGAA
Q6759Q6760	IFITM3	Interferon induced transmembrane protein 3	F	TGAAGTCTAGGGACAGGAAGA
			R	CATGAGGATGCCCAGAATCA
Q6883Q6884	SARS-CoV-2 Spike		F	CAATGGTTTAACAGGCACAGG
			R	CTCAAGTGTCTGTGGATCACG
Q6906Q6907	SARS-CoV-2 N 1 CDC		F	GACCCCAAAATCAGCGAAAT
			R	TCTGGTTACTGCCAGTTGAATCTG
Q6908Q6909	SARS-CoV-2 N 2 CDC		F	TTACAAACATTGGCCGCAAA
			R	GCGCGACATTCCGAAGAA
